# The Subject of Criminal Abortion Discussed in a Religious Newspaper

**Published:** 1867-03

**Authors:** 


THE SUBJECT OF CRIMINAL ABORTION DISCUSSED IN A RELIGIOUS NEWSPAPER. The *Northwestern Christian Advocate* devotes one entire page to a full discussion of the evils of abortion, in its issue of March 13th. It is an indication of honesty, and earnestness which we had not expected to see. This is the first instance, to our knowledge, of the subject being freely and fearlessly discussed in a public paper, and in this instance nothing has been omitted or covered; it is truly creditable to both the head and heart of the author. It will be again noticed when we have more space to devote to the subject, as we have one or two items to suggest.

